# Postnatal care in crisis: utilization and determinants during the Tigray conflict (2023)

**DOI:** 10.1186/s12978-026-02311-2

**Published:** 2026-03-27

**Authors:** Asfawosen Aregay, Brhane Ayele, Mussie Alemayehu, Mulugeta Woldu, Hailay Gebretnsae, Tsegay Hadgu, Mulugeta Tilahun, Ataklti Gebretsadik, Fana Gebreslassie, Kiros Demoz, Tsegay Wellay, Znabu Hadush, Haftu Gebrehiwot, Desalegn Meresa, Reda Shamie, Assefa Ayalew, Adhena Ayaliew, Mebrahtu Kalayu, Gebrehaweria Gebrekurstos, Liya Mamo, Tadele Tesfean, Ferehiwot Hailemariam, Abraham Gebrelibanos, Hayelom Kahsay, Afework Mulugeta, Abraham Aregay

**Affiliations:** 1 Tigray Health Research Institute (THRI), Mekelle, Tigray Ethiopia; 2 Mekelle University College of Health Sciences (MUCHS), Mekelle, Tigray Ethiopia; 3 Tigray Regional Health Bureau (TRHB), Mekelle, Tigray Ethiopia; 4 MARCH Research Center, Mekelle University, Mekelle, Tigray Ethiopia; 5 University of Florence and University of Palermo, Palermo, Italy

**Keywords:** Postnatal care, Utilization, Maternal health, Newborn health, Tigray conflict, Determinants

## Abstract

**Background:**

Tigray’s previously strong health system and high postnatal care (PNC) utilization were devastated by the November 2020 conflictct. This brought drastically increased maternal and newborn mortality rates, exceeding national figures, and this also led to a lack of evidence on current PNC service use. This study therefore aims to assess the extent of PNC service utilization and its determinants among mothers who gave birth during the conflict.

**Methods:**

A community-based, cross-sectional study was conducted, using multi-stage cluster sampling to select 3,747 participants aged 15–49 from 19 districts. Continuous variables were described using means and standard deviations, while categorical variables were described using frequencies and percentages. To identify factors influencing postnatal care utilization, count regression models (Negative binomial, Modified poisson, and Quasi poisson) were employed in STATA version 14. The analysis used a 95% confidence interval, and a *p*-value < 0.05 was used to declare its statistical significant.

**Results:**

This study found a low postnatal care utilization rate of only 20% (95% CI: 18.7, 21.3) among participants for their recent births. Several factors were identified as determinants of this low utilization. Women who attended antenatal care (aPR = 1.6, 95% CI: 1.18, 2.16) and delivered at a health facility (aPR = 3.7, 95% CI: 3.05, 4.47) were more likely to utilize postnatal care. Conversely, women with limited transport access (aPR: 0.77, 95% CI: 0.64, 0.94), those in host communities (aPR: 0.64, 95% CI: 0.51, 0.8), and rural residents (aPR: 1.53, 95% CI: 1.2, 1.96) were less likely. Younger age, occupation, and administrative zone also influenced postnatal care utilization.

**Conclusion:**

The conflict in Tigray is associated with a catastrophic decline in postnatal care (PNC) utilization, which dropped from 81 to 20%. With only 14.5% of women completing recommended four visits, mothers and neonates face significantly heightened health risks. Our findings suggest this decline is linked to facility destruction, displacement, and transport barriers, alongside demographic factors like urban residence and unemployment. These results underscore the urgent need for targeted interventions, including infrastructure renovation and integrated PNC education. Addressing these multifaceted barriers is likely essential to restoring service uptake and maternal health outcomes.

## Abstract (plain language summary)

This study examined postnatal care (PNC) use among 3,747 women in Tigray, Ethiopia, during the recent conflict. They found a shockingly low PNC utilization rate of only 20%, a steep decline from pre-conflict rates. The war devastated the region’s healthcare system, with widespread damage to facilities and a mass exodus of healthcare workers. This drastically limited access to care. The study identified several factors associated with PNC use. Women with better transport access were more likely to receive PNC. Internally displaced women used PNC more than host community women, possibly due to targeted aid. Rural women surprisingly used PNC more than urban women, likely due to mobile clinics operating in rural areas. Women who had more antenatal care visits and those who delivered in health facilities were also much more likely to receive PNC. Younger women and women employed by the government were also more likely to use PNC.

The conflict created immense barriers to PNC, including fear and insecurity, damaged infrastructure, displacement, and a breakdown of the health system. The study highlights the urgent need to rebuild the health system, improve access to care, and provide targeted education about PNC, especially for urban, displaced, and older women. The researchers acknowledge limitations, including potential recall bias and the exclusion of some areas due to security concerns.

## Background

The World Health Organization (WHO) defines the postnatal period as the six weeks following childbirth [1]. This period is critical for both mother and child, as the majority of maternal and neonatal deaths occur within it [2]. Notably, nearly half of all maternal deaths are reported to happen within the first week postpartum [1]. WHO recommends four postnatal care visits: within 24 h of birth, two to three days postpartum, six to seven days postpartum, and at six weeks [1]. Mothers and their families should be aware of potential postpartum danger signs, including unexpected vaginal bleeding, difficulty breathing, fever, severe headaches, redness, shortness of breath, or chest pain [3]. Therefore, providing proper postnatal care services by skilled health personnel is essential for saving the lives of both mother and newborn [4]. This care also offers an opportunity for health professionals to identify, monitor, and manage any health conditions that may develop in either the mother or newborn during this critical period [5].

Efforts to improve maternal health are often hindered by various factors, with conflict being a significant impediment as noted by the United Nations (UN) [6]. Violence and instability can disrupt governmental and international aid, further impeding health promotion. Numerous studies have shown that postnatal care (PNC) service utilization is influenced by factors such as maternal age, occupation, religion, marital status, antenatal care visits, delivery location, education level, wealth index, birth order, and media exposure [4, 7–10]. In conflict environments severely undermine healthcare delivery through systemic and individual barriers. Key challenges include supply chain disruptions that deplete essential resources and strained human capital due to difficulties in staff retention. Additionally, physical infrastructure damage restricts geographical access, while heightened security risks create psychological deterrents that prevent patient care-seeking behavior [11].

In low-income countries, a significant proportion of women face complications after childbirth, with nearly 40% experiencing some form of complication and 15% developing life-threatening conditions [12]. Ethiopia, a sub-Saharan African nation, is among the six countries that account for approximately half of all global maternal deaths [13]. Despite global progress in reducing maternal and child mortality rates, Ethiopia’s rates remain high. The country’s maternal mortality ratio stands at 412 per 100,000 live births, while under-five, infant, and neonatal mortality rates are 67, 48, and 29 per 1000 live births, respectively [7].

Prior to the conflict, the Tigray region of Ethiopia had a relatively well-developed health system [14–16], which served as a benchmark for Ethiopia’s flagship health extension program [17, 18]. Studies indicated higher rates of postnatal care utilization in Tigray compared to the national average. For instance, nationally, Ethiopia achieved PNC rates of approximately 33.8% by 2019 [19]. More strikingly, the Tigray region itself reported significantly higher utilization, with figures as high as 62.9% in 2019 [19] and even 81% prior to the conflict [20, 21].

The trajectory of healthcare in Tigray was severely interrupted by an armed conflict that began in November, 2020 and lasted until a peace agreement was signed in November, 2022 [22, 23]. This two-year conflict resulted in a catastrophic humanitarian crisis and the widespread destruction of the region’s health system. Estimates indicate that 70% to 90% of health facilities were damaged or rendered non-functional [14, 15, 24].

Moreover, data from the post-war period indicates a dramatic decline in health outcomes within Tigray, where maternal and infant mortality rates have significantly exceeding the national averages [25]. Although existing research highlights general determinants of postnatal care (PNC) usage such as education, financial status, and access to information [4, 7–10], there is a significant gap in understanding how these variables function when an entire healthcare system has collapsed.

Understanding the factors influencing PNC utilization is crucial in conflict-affected contexts like Tigray. Given the severe departure from pre-conflict service levels, there is an urgent need to evaluate the current state of maternal health services. This study aims to assess the extent of PNC service utilization and its determinants among mothers who gave birth during the conflict in Tigray, providing the evidence base necessary for targeted recovery efforts.

## Methods

### Study area, population, sample size, sampling and data collection

**Fig. 1:**
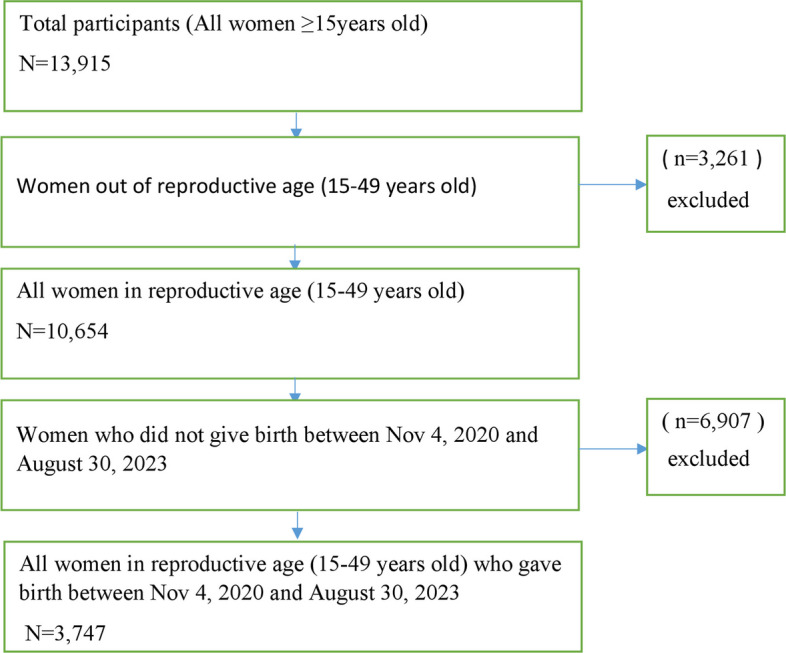
Sampling procedure for postnatal care utilization assessment in the Tigray region (2023)

The study was conducted in the Tigray region of northern Ethiopia [26], which has a total population of 7,969,000 [27] and healthcare infrastructure comprised two specialized comprehensive hospitals, 14 general hospitals, 24 primary hospitals, 231 health centers, and 743 health posts [26, 28]. In 2023, an integrated post-conflict survey was conducted in the Tigray region, encompassing 13,819 households and 10,654 women of reproductive age, and this study was part of the integrated survey [26]. For this study, a sample was randomly selected from 19 districts (out of the region’s seven zones). A multi-stage cluster sampling method was employed to select these 51 kushets and 6 internally displaced person (IDP) sites based on a stratified random selection considering rural-to-urban ratios for districts, and purposive selection of key IDP sites to ensure representation. Due to security concerns, the entire western zone and parts of the northwest, central, eastern and southern zones were excluded from the sampling frame [26]. Data collection took place from August 1 to 30, 2023. All live births (*N* = 3,747) occurring prior to August 30, 2023, were included in the current study (Fig. 1).

### Study variables

The study’s outcome variable was PNC service utilisation which was considered as at least one postnatal check for both the mother and the neonate within 42 days of childbirth. This was measured as a count data variable, meaning it could only take whole number values. In this case, the count ranged from 0 to 4, likely representing the number of postnatal care visits a woman made. So, a woman could have had 0 visits, 1 visit, 2 visits, 3 visits, or 4 visits.

#### Independent variables

The study examined several factors that might influence whether women in Tigray, Ethiopia used postnatal care services. These factors, called independent variables, were chosen based on previous research [9, 26, 29]. They can be grouped into a few categories:

##### Socio-demographic factors

These describe the women and their households, including their age, marital status, religion, place of residence, the zone they live in, the education level of both the woman and her husband, the household’s income, and the woman’s occupation.

##### Obstetric history and related factors

These relate to the woman’s pregnancy and childbirth experiences, including whether she attended antenatal care appointments, where she gave birth, and the number of under five children she has.

##### Other factors

These include access to transportation to health facilities, whether the woman has access to electronic media (like radio or television), and her status as a local resident or internally displaced person.

### Data management and analysis

We used descriptive statistics to summarize our data. For continuous variables like age and postnatal care visit, we calculated the mean and standard deviation. For categorical variables like marital status, we used frequencies and percentages. These results were presented in tables, graphs, and text. For our main analysis, we used a 95% confidence interval and considered results statistically significant if the *p*-value was less than 0.05. The statistical software STATA version 14 was used. Because our outcome variable (number of postnatal care visits) was a count (0, 1, 2, 3, or 4), we initially planned to use a Poisson regression model. However, our data showed overdispersion (meaning the variability was higher than expected for a Poisson model) [30].

### Model selection

Therefore, after considering several options, we determined that a Negative Binomial regression model was the best fit for our data (Table 1).

**Table 1:** Model selection: comparison of negative binomial, quasi-poisson, and modified poisson regression for postnatal care utilization

Models	AIC	BIC	Log-likelihood
Negative binomial count regression	3,314.9	3,449.9	−1,633.5
Modified poisson regression	3,701.1	3,815.2	−1,830.6
Quasi poisson regression	3,701.1	3,815.2	−1,830.6

^**^AIC akaike information criterion, *BIC* bayesian information criterion

Poisson regression is unsuitable for handling overdispersed count data, where the variance exceeds the mean [31]. Therefore, three models Negative Binomial, Quasi-Poisson, and Modified Poisson regressions were assessed and compared [32] to determine the best fit for this type of data.

To identify the best-fitting model, model diagnostics were performed, and the fitness of Negative Binomial, Quasi-Poisson, and Modified Poisson regression models was compared using criteria such as log-likelihood, the Akaike Information Criterion (AIC), and the Bayesian Information Criterion (BIC) [33]. These criteria are essential for selecting the optimal model while minimizing the risk of overfitting [34, 35]. The model with the lowest AIC and BIC values, and the highest likelihood ratio, was selected.

## Results

### Socio demographic and economic characteristics of the study participants

A total of 3,747 women aged 15–49 participated in the study. The average age of the respondents was 29.4 years (± 6.4). Nearly half (49.7%, *n* = 1,863) were between 25 and 34 years old. The majority of participants were rural residents (78.7%, *n* = 2,948), married (91.1%, *n* = 3,415), and housewives (58.5%, *n* = 2,190). Regarding education, 42.2% (*n* = 1,580) were unable to read and write, 32.1% (*n* = 1,279) had completed primary education, and 23.7% (*n* = 888) had secondary education or above (Table 2).

**Table 2:** Socio-demographic and economic characteristics of women utilizing postnatal care during the Tigray conflict ((*N* = 3,747)

Variables	Categories	Frequencies	Percentages
Age (in years)	15–24	891	23.8
	25–34	1,863	49.7
	35–49	993	26.5
Women’s occupation	Housewife	2,190	58.5
	Farmer	1,181	31.5
	Others	376	10.0
Women’s education	Unable to read and write	1,580	42.2
	Primary	1,279	32.1
	Secondary and above	888	23.7
Husband’s education	Unable to read and write	1,126	32.7
	Primary	1,491	43.3
	Secondary and above	826	24.0
Religion	Orthodox	3,665	97.8
	Muslim	82	2.2
Marital status	Married	3,415	91.1
	Others	332	8.9
Wealth status of household	Poor	2,237	59.7
	Medium	1,466	39.1
	Rich	44	1.2
Residence	Urban	799	21.3
	Rural	2,948	78.7
Mobile phone ownership	No	1,429	38.1
	Yes	2,318	61.9
Displacement status	Host community	3,288	87.8
	IDP	459	12.2
Administrative zones	Mekelle	358	9.6
	Central	978	26.1
	Eastern	676	18.0
	Northwest	800	21.4
	Southern	680	18.2
	Southeast	255	6.8

*IDP* Internal displaced people

### Postnatal care utilization and women’s health related characteristics

A large majority of the women in the study (72.3%, 95% CI: 70.8, 73.7) were married before the age of 18, and an even greater proportion (91.3%, 95% CI: 90.4, 92.2) had three or more children. A 62.2% of the women (62.2%, 95% CI: 60.6, 63.7) attended antenatal care services during their most recent pregnancy, while 38% (38.8%, 95% CI: 37.2, 40.4) gave birth in a health facility.

This study found that only 1 in 5 women (20%, 95% CI: 18.7, 21.3%) utilized PNC services for their recent births. This highlights a critical gap in maternal healthcare; while a small portion of women accessed care, there is a serious scarcity of PNC use, particularly regarding adherence to multiple visits. Among those who received PNC, 57.1% had only one visit, while only 14.5% reached the recommended three or more visits, indicating that even when care is initiated, consistent follow-up is rarely achieved (Table 3).

**Table 3:** Postnatal care utilization and women’s health characteristics during the Tigray conflict (2023)

Variables	Categories	Frequencies	% and 95% CI
Women who attend PNC	Yes	749	20 (18.7–21.3)
	No	2,998	80 (78.7–81.3)
Number of PNC visits	Once	428	57.1 (53.5–60.7)
	Twice	212	28.3 (25.1–31.7)
	Three times	109	14.6 (12.1–17.3)
ANC follow up visits	Yes	2,330	62.2 (60.6–63.7)
	No	1,417	37.8 (36.3–39.4)
Number of ANC visits	Once	341	14.6 (13.2–16.1)
	Twice	575	24.7 (22.9–26.5)
	Three times	611	26.2 (24.5–28.1)
	Four or above	803	34.5 (32.5–36.4)
Number of under five children	≤ 2	326	8.7 (7.8–9.7)
	3 and above	3,421	91.3 (90.4–92.2)
Age at marriage (years)	< 18	2,663	72.3 (70.8–73.7)
	≥ 18	1,021	27.7 (26.3–29.2)
Place of delivery	Health facilities	1,453	38.8 (37.2–40.4)
	Home/other places	2,294	61.2 (59.6–62.8)
Distance to the nearest HFs	< 1 h	2,208	58.9 (57.3–60.5)
	≥ 1 h	1,539	41.1 (39.5–42.7)
Transport access to HFs	No	1,422	38.0 (36.4–39.5)
	Yes	2,325	62.0 (60.5–63.6)

*HFs* Health facilities, *ANC* Antenatal care, *PNC* Postnatal care, *CI* Confidence interval, *hr* Hour

### Factors limiting postnatal care service utilization during the Tigray conflict

**Fig. 2:**
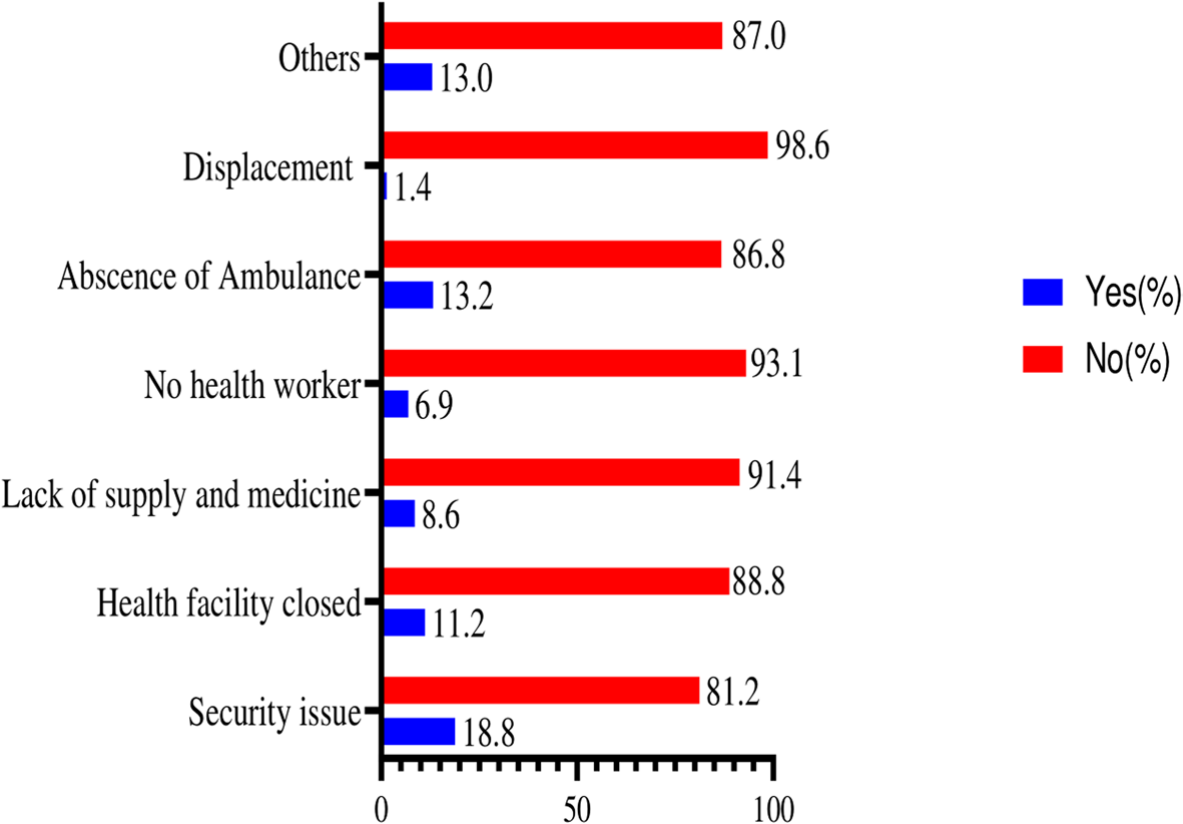
Barriers to postnatal care service utilization during the conflict in the Tigray region

Figure 2 showed that the conflict in Tigray created a cascade of obstacles preventing women from accessing postnatal care (PNC). First, the conflict itself generated pervasive fear and insecurity, deterring women from traveling to health facilities due to violence, active fighting, and movement restrictions [36–38]. Simultaneously, the conflict devastated the region’s healthcare infrastructure, with facilities damaged or destroyed and those remaining often lacking essential supplies and staff [15, 24].

The conflict also led to a mass displacement of people, creating further barriers for women seeking care, including lack of transportation, resources, and information. Transportation networks were severely compromised, with damaged roads and non-existent ambulance services, making emergency care nearly impossible to obtain. Exacerbating these issues, the conflict produced a significant breakdown of the health system, even where facilities remained, leading to fragmented and disorganized care [28, 39–41].

Finally, the flight of healthcare workers due to safety concerns and the disruption of their ability to reach workplaces created a severe shortage of medical personnel. These interwoven factors combined to drastically limit women’s access to PNC during the Tigray conflict (Fig. 2).

### Factors associated with postnatal care utilization

After controlling for other factors using Negative Binomial Poisson regression, several predictors for postnatal care service utilization were identified: transport access, administrative zone, displacement status, antenatal care attendance, place of delivery, residence, age, and occupation.

Women with limited transport access to the nearest health facility were 23% less likely to utilize PNC services (aPR: 0.77, 95% CI: 0.64, 0.94) compared to those living closer. Women from host communities were 36% less likely to use PNC services than internally displaced women (aPR: 0.64, 95% CI: 0.51, 0.8). Women in the Mekelle zone were 40% more likely to utilize PNC services compared to those in the southern zone (aPR: 1.4, 95% CI: 1.03, 1.91). Contrary to other studies, rural residents were 53% *more* likely to utilize PNC services than urban residents (aPR: 1.53, 95% CI: 1.2, 1.96).

Women working in government institutions were 54% more likely to receive PNC services (aPR: 1.54, 95% CI: 1.07, 2.23), while self-employed (daily laborers, merchants etc.) were 40% less likely (aPR: 0.6, 95% CI: 0.45, 0.81) compared to housewives. Women aged 15–24 were 30% more likely to receive PNC services compared to women aged 35–49 (aPR: 1.30; 95% CI: 1.02, 1.65).

Finally, women with four or more ANC visits were 60% more likely to utilize PNC services (aPR = 1.6, 95% CI: 1.18, 2.16), and women who delivered at health facilities were 3.7 times more likely to utilize PNC services (aPR = 3.7, 95% CI: 3.05, 4.47) (Table 4).

**Table 4:** Factors affecting postnatal care utilization during the Tigray conflict (2023)

Variables	Category (*N* = 3747)	uPR (95% CI)	aPR (95% CI)
Age (in years)	15–24	1.49 (1.21, 1.84)^***^	1.30 (1.02, 1.65)^*^
	25–34	0.87 (0.73, 1.04)	1.08 (0.88, 1.33)
	35–49	1	1
Occupation of women	Housewife	1	1
	Government employee	1.80 (1.33, 2.44)^***^	1.54 (1.07, 2.23)^*^
	Farmer	0.51 (0.42, 0.62)^***^	0.93 (0.74, 1.18)
	Self-employed/Merchant/Daily labor	1.02 (0.78, 1.33)	0.60 (0.45, 0.81)^***^
Place of delivery	Health facilities	5.00 (4.15, 6.02)^***^	3.69 (3.05, 4.47)^***^
	Home	1	1
Number of ANC attendance	Once	1	1
	Twice	1.54 (1.05, 2.25)^*^	1.33 (0.97, 1.83)
	Three times	1.81 (1.26, 2.62)^**^	1.27 (0.93, 1.72)
	Four or above	3.06 (2.16, 4.34)^***^	1.60 (1.18, 2.16)^**^
Displacement status	Host	0.54 (0.44, 0.65)^***^	0.64 (0.51, 0.80)^***^
	IDP	1	1
Place of residence	Rural	0.60 (0.51, 0.70)^***^	1.53 (1.20, 1.96)^***^
	Urban	1	1
Administrative zones	Southern	1	1
	Southeast	1.01 (0.77, 1.34)	0.93 (0.67, 1.29)
	Mekelle	1.74 (1.34, 2.25)^***^	1.40 (1.03, 1.91)^*^
	Eastern	0.92 (0.71, 1.19)	0.93 (0.70, 1.23)
	Central	0.78 (0.61, 1.00)	0.79 (0.60, 1.04)
	Northwest	0.69 (0.53, 0.91)	0.81 (0.60, 1.11)
Transport access to HFs	Yes	1	1
	No	0.54 (0.47, 0.63)^***^	0.77 (0.64, 0.94)^**^

*uPR* unadjusted Prevalent rate, *aPR* adjusted Prevalent Rate, *CI* Confidence interval, *ANC* antenatal care, *PNC* Postnatal care, *IDP* Internal displaced people, *HF* Health facilities

^***^P < 0.001, ^**^P < 0.01, *P < 0.05

## Discussion

The observed 1 in 5 (20%) of PNC utilization stands in stark contrast to the pre-conflict context. Prior to the war, Tigray boasted high maternal health service utilization, reaching as high as 81% [19–21, 42]. This dramatic reduction to merely 20% unequivocally illustrates the devastating consequences of the conflict and the total disruption of healthcare access pathways, a decline that far exceeds what is typically expected even in low-resource settings [41, 43, 44].

This data highlights a critical gap in maternal healthcare; while a small fraction of women accessed care, there is a severe scarcity of sustained PNC use, particularly regarding adherence to multiple visits. Among those who received any care, 57.1% had only a single visit, while only 14.5% reached the recommended threshold of three or more visits. This reveals a significant drop-off effect; even when women successfully navigate the system for an initial visit, they face considerable gaps to completing the full continuum of care.

Several factors explain this disparity. The conflict created a cascade of obstacles: pervasive fear and insecurity deterred women from traveling, while the destruction of infrastructure and the mass exodus of healthcare workers led to a functional breakdown of the health system [15, 24, 36–38].

Geographical barriers further exacerbated these issues. This study found that women with limited transport access were 23% less likely to utilize PNC services than those living closer to health facilities, a finding consistent with data from Tanzania and other parts of Ethiopia [45, 46]. Regionally, women in the Mekelle zone showed higher utilization than those in the Southern zone. This disparity stems directly from the war’s disproportionate impact: while 78.6% of facilities in Mekelle remained functional, only 14.6% in the Southern zone were operational due to widespread looting and destruction (affecting 70–80% of facilities region-wide) [28, 41].

Interestingly, host community mothers were 36% less likely to utilize PNC than internally displaced women (IDPs). This is likely because humanitarian organizations prioritized services specifically for IDP populations [14]. Furthermore, in a counterintuitive finding, rural residents were 1.54 times more likely to utilize PNC services than urban residents. This difference can be attributed to the presence of Médecins Sans Frontières (MSF) mobile clinics operating in rural areas and IDP sites where the formal health system had collapsed [16]. However, it is crucial to note that many remote rural areas remained unreachable by any organization, which explains why this finding contrasts with studies in Uganda and other regions of Ethiopia [47, 48]. These discrepancies likely arise from differences in study populations, time periods, sample sizes, and socio-cultural practices, highlighting the need for further research to explore these variations.

Socio-economic status also played a pivotal role. Government-employed women were more likely to utilize PNC services than housewives, while self-employed women (daily laborers and merchants) were the least likely to seek care. This aligns with findings from Nepal and Ethiopia [49, 50], suggesting that economic independence and higher financial status empower women to prioritize healthcare decisions [50, 51]. Similarly, younger women aged 15–24 showed a higher propensity for PNC utilization than older women 35–49, possibly due to higher health literacy and a greater likelihood of adopting new health behaviors [52]. However, this result contrasts with studies conducted in Ghana’s Savannah region and Ethiopia’s East Amhara region [53, 54], suggesting that age-related PNC utilization patterns can vary across different contexts.

Finally, the study reinforces the importance of the continuum of care. Women who attended four or more Antenatal Care (ANC) visits or delivered at a health facility were significantly more likely to receive PNC. This underscores the strong link between professional clinical contact, maternal counseling, and subsequent service uptake [7, 8, 12, 45, 49, 55].

### Strengths and limitations

This study provides a robust quantitative analysis of maternal healthcare collapse during the Tigray conflict, utilizing a large sample (*N* = 3,747) and rigorous Negative binomial regression to accurately handle overdispersed count data. A key strength is the inclusion of both host community and displaced populations, offering a rare comparative view of service utilization in a humanitarian crisis.

However, several limitations warrant caution. The retrospective design may introduce recall bias regarding healthcare visits occurring up to two years prior. Furthermore, the cross-sectional nature of the data identifies significant associations but precludes the establishment of definitive causal links. Most critically, active conflict necessitated the exclusion of the Western zone and parts of the Northwest, Central, Eastern, and Southern zones, potentially limiting the generalizability of the findings to the most volatile zones. Additionally, the observed rural PNC utilization advantage likely reflects the localized presence of mobile clinics at the time of the survey rather than a systemic trend. These factors must be considered when interpreting the results within the broader context of the region’s healthcare recovery.

## Conclusions

The conflict in Tigray is associated with a catastrophic decline in postnatal care (PNC) utilization, which dropped from 81% to 20%. With only 14.5% of women completing recommended four visits, mothers and neonates face significantly heightened health risks. Our findings suggest this decline is linked to facility destruction, displacement, and transport barriers, alongside demographic factors like urban residence and unemployment. These results underscore the urgent need for targeted interventions, including infrastructure renovation and integrated PNC education. Addressing these multifaceted barriers is likely essential to restoring service uptake and maternal health outcomes.

## Data Availability

The datasets used and/or analyzed during the current study are available from the corresponding author on reasonable request.

## Abbreviations

AMRIF: Africa Medical Research Fund
ANC: Antenatal care
aPR: Adjusted Prevalence Ratio
CSA: Central Statistical Agency
IDP: Internal Displaced People
IRB: Institutional review board
MUCHS: Mekelle University College of Health Science
PNC: Postnatal Care
SD: Standard Deviation
THRI: Tigray Health Research Institute
RHB: Regional Health Bureau
UNICEF: United Nation Children Fund
UNFPA: United Nations Population Fund
uPR: Unadjusted Prevalence Ratio
WHO: World Health Organization
